# Zooming in on Cerebral Amyloid Angiopathy in the Context of Amyloid-Related Imaging Abnormalities

**DOI:** 10.1093/geroni/igaf122.1006

**Published:** 2025-12-31

**Authors:** Mo-Kyung Sin, Yuan Luo

## Abstract

The significance of cerebral amyloid angiopathy (CAA) has been heightened since anti-amyloid therapies were approved. Amyloid-related imaging abnormalities (ARIA) are a major side effect of anti-amyloid therapies, and CAA is the major risk factor for ARIA. CAA is highly prevalent in Alzheimer’s disease, and both are driven by impaired amyloid β clearance. In addition, the imaging and pathological manifestations of ARIA are similar to CAA, supporting both sharing a common pathway. High prevalence of CAA in AD places many patients at risk for ARIA-associated morbidity and high cost for health care. ARIA can be life-threatening in about 1% of patients, highlighting the need for better understanding of CAA. Thus, we assembled nationally and internationally well-known experts on CAA and Alzheimer’s disease and related dementias research to cover the CAA accumulation pathway, risk factors and consequences of CAA in community-based clinical pathologic studies, vascular considerations for patients receiving anti-amyloid therapies, and general risk factor and management strategies. Our first presenter, Dr. Donna Wilcock will present on the time course of CAA progression in the APPSw mouse model, 2nd presenter, Dr. David Bennett on CAA risk factors and consequences from the Religious Orders Study and Rush Memory and Aging Project, and the Pathology, Alzheimer’s and Related Dementias Study, 3rd presenter, Dr. Steve Greenberg on the key role of vasculature in generating ARIA, and our last presenter, Dr. Edward Zamrini on general CAA risk factors and management strategies. We believe this symposium has timely and real-life clinical and research implications.

